# Outcomes after coronary artery bypass grafting and percutaneous coronary intervention in diabetic and non-diabetic patients

**DOI:** 10.1093/ehjqcco/qcab065

**Published:** 2021-09-07

**Authors:** Hanna-Riikka Lehto, Klas Winell, Arto Pietilä, Teemu J Niiranen, Jyri Lommi, Veikko Salomaa

**Affiliations:** Department of Public Health, University of Turku, 20014 Turku, Finland; THL – Finnish Institute for Health and Welfare, Department of Chronic Disease Prevention, P.O. Box 30, 00271 Helsinki, Finland; THL – Finnish Institute for Health and Welfare, Department of Chronic Disease Prevention, P.O. Box 30, 00271 Helsinki, Finland; THL – Finnish Institute for Health and Welfare, Department of Chronic Disease Prevention, P.O. Box 30, 00271 Helsinki, Finland; THL – Finnish Institute for Health and Welfare, Department of Chronic Disease Prevention, P.O. Box 30, 00271 Helsinki, Finland; Department of Medicine, Turku University Hospital and University of Turku, P.O. Box 52, 20521 Turku, Finland; Heart and Lung Center, Cardiology Division, P.O. Box 340, 00029 HUS, Helsinki, Finland; THL – Finnish Institute for Health and Welfare, Department of Chronic Disease Prevention, P.O. Box 30, 00271 Helsinki, Finland

**Keywords:** Coronary heart disease, Diabetes, Coronary artery bypass surgery, Percutaneous coronary intervention, Prognosis, Survival

## Abstract

**Aims:**

To assess the prognosis of patients with coronary heart disease (CHD) after first myocardial revascularisation procedure in real-world practice and to compare the differences in outcomes of coronary artery bypass grafting (CABG) and percutaneous coronary intervention (PCI) among diabetic and non-diabetic patients.

**Methods and results:**

A database was compiled from the national hospital discharge register to collect data on all cardiac revascularisations performed in Finland in 2000–2015. The outcomes (all-cause deaths, cardiovascular (CV) deaths, major CV events and need for repeat revascularisation) after the first revascularisation were identified from the national registers at 28 day, 1 year, and 3 year time points.

A total of 139 242 first-time revascularisations (89 493 PCI and 49 749 CABG) were performed during the study period. Of all the revascularised patients, 24% had diabetes, and 76% were non-diabetic patients. At day 28, the risk of fatal outcomes was lower after PCI than after CABG among non-diabetic patients, whereas no difference was seen among diabetic patients. In long-term follow-up the situation was reversed with PCI showing higher risk compared with CABG for most of the outcomes. In particular, at 3 year follow-up the risk of all-cause deaths was elevated among diabetic patients [HR 1.30 (95% CI 1.22–1.38) comparing PCI with CABG] more than among non-diabetic patients [HR 1.09 (1.04–1.15)]. The same was true for CV deaths [HR 1.29 (1.20–1.38) among diabetic patients, and HR 1.03 (0.98–1.08) among non-diabetic patients].

**Conclusion:**

Although PCI was associated with better 28 day prognosis, CABG seemed to produce better long-term prognosis especially among diabetic patients.

## Graphical Abstract

**Graphical Abstract:**
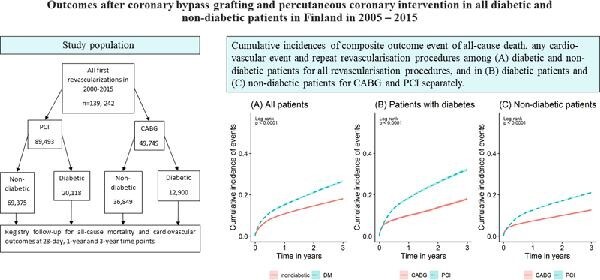

## Introduction

Globally, approximately 463 million adults are estimated to have diabetes. The prevalence of diabetes has been steadily increasing over the past few decades, and the global prevalence has been projected to further increase by 51% by the year 2045.^1^ The incidence of coronary heart disease (CHD) is higher among patients with diabetes than in the general population, and cardiovascular diseases (CVDs), mainly CHD, are the most common cause of death among diabetic patients.^2–4^ Diabetes is known to cause more generalized, diffuse atherosclerosis, and consequently, multivessel coronary artery disease (CAD) is detected more often among patients with diabetes than in normoglycemic subjects.^2^,^5–8^

Survival after revascularisation is known to be worse for patients with diabetes than in patients without diabetes.^9^ Hence, the choice of optimal revascularisation method for diabetic patients with CHD has been debated over the past few decades, and numerous randomized controlled trials (RCTs) have been carried out to compare the outcomes of coronary artery bypass grafting (CABG) and percutaneous coronary intervention (PCI).^9–14^ In diabetic patients with multivessel CAD, CABG has been shown to associate with better long-term survival compared to PCI with bare metal stents (BMSs)^9^ as well as with drug eluting stents (DESs).^10–14^ However, when analyzing a composite outcome of death, myocardial infarction (MI) and stroke among diabetic patients with 1- or 2-vessel disease,^14^ or when analyzing individual components of a composite outcome measure among diabetic patients with left main CAD or multivessel disease,^15^ no long-term differences have been observed between CABG and PCI. Furthermore, PCI has challenged CABG with its wider availability and lower risk for stroke, and with the development of DESs.^10,16,17^ In an RCT comparing CABG and PCI with a newer generation everolimus-eluting stent, no difference was observed in the composite outcome of death, MI and stroke between these methods among diabetic patients with low or intermediate complexity left main CAD at 3 years.^18,19^

The latest recommendation in the European clinical practice guidelines is that in less complex, 1- to 2-vessel disease without left anterior descending (LAD) coronary artery involvement, PCI is recommended; whereas with LAD disease, both revascularisation methods can be implemented. Further, in intermediate or high complexity LAD and 3-vessel CAD, CABG is recommended for patients with diabetes.^20^

The aforementioned RCTs provide rather clear evidence for choosing the treatment strategies. However, due to the specific inclusion criteria used in RCTs, the generalizability of their results into real-world practice can be limited. Furthermore, by using real-world data from the hospital discharge register (HDR), we have previously shown that PCI was the more commonly used revascularisation procedure among both diabetic and non-diabetic patients.^21^ Thus, the aim of this study was to compare the prognosis among diabetic and non-diabetic patients with CHD after the first revascularisation procedure, either CABG or PCI, and to compare the differences in the short- and long-term outcomes between the two revascularisation methods in real-world practice.

## Methods

### Study design and data sources

We used the nationwide HDR to identify all patients who had their first cardiac revascularisation carried out in Finland during the years 2000–2015 (Supplementary Figure S1). The data on revascularisations has been collected using electronic templates filled out by the treating physician for every invasive cardiac procedure performed.

The HDR and Finnish Drug Reimbursement Register (DRR) were used to identify diabetes and other pre-existing cardiovascular comorbidities. The outcomes were identified in the HDR and causes of death register (CDR). Registration in these electronic health care registers is mandatory by law, and these registers cover the whole country. They have been described in detail in earlier publications.^21,22^

The outcomes after the first revascularisation were evaluated at the time points of 28 days, 1 year, and 3 years. If repeat revascularisations were carried out within 28 days from the incident procedure, these patients were excluded from the 1 year and 3 year analyses regarding the repeat revascularisations. Finally, we further analyzed all the other 1 and 3 year outcomes separately for those patients who had an event-free 28 day survival.

### Definitions of revascularisation procedures

Information on the revascularisation procedures is recorded using codes defined by the Nordic Medico-Statistical Committee (NOMESCO) from 1996 onwards. Coronary artery bypass grafting codes collected for the study were FNA, FNB, FNC, FND, and FNE; and PCI codes FN1AT, FN1BT, FN1YT, FNF, FNG, and TFN40 (for detailed description of the NOMESCO codes, see Supplementary Table S1). If CABG followed PCI within 7 days, then CABG was considered to be the first revascularisation overriding the PCI procedure. A procedure was defined urgent if it was necessary to be performed as an emergency procedure or within 7 days after hospitalization. After that the procedure was considered elective.

### Definitions of diabetes and pre-existing comorbidities

The diagnoses in the HDR are recorded according to the International Classification of Diseases (ICDs) codes as defined in the Finnish version of ICD-10 applied from 1996 onwards. To better capture the diabetic patients and the comorbidities of all patients, we also used data on special reimbursements from the Finnish DRR.

A patient was considered to have diabetes if she/he had either an ICD-10 code E10 or E11 in the HDR, or was entitled to special reimbursements for hypoglycaemic medications in the DRR. A patient was considered to have hypertension if she/he had an ICD-10 code I10-I13 and I15 in the HDR or was entitled to special reimbursements for antihypertensive medications; and to have chronic heart failure (CHF) if she/he had the ICD-10 code I11 or I50 in the HDR, or was entitled to special reimbursements for CHF medications. Previous MI, CHD, or cardiomyopathy were identified with ICD-10 codes I21–I23, I25, I42–I43, respectively, in the HDR. Valvular defects were recognized based on the ICD-codes I05–08, I34–37, I39.0–I39.4, and atrial fibrillation on ICD-10 code I48. Previous strokes were identified with ICD-10 codes I61 and I63 (excluding codes I63.6, I64, and I60.0–I60.9) and peripheral artery disease (PAD) with the ICD-code I70.2. The used ICD-10 codes are described in detail in Supplementary Table S2.

### Definitions for outcome events during follow-up

To obtain data on fatal cases, causes of death were collected from the CDR. A death was considered to be due to CVD if the cause of death was determined by any of the following ICD-10 codes in the CDR: I20–I25, I61–I64, R96, or R98 (Supplementary Table S2).

For cause-specific outcomes, ACS were identified from electronic health registers using the ICD-codes I20.0, I21, and I22 (Supplementary Table S2). Stroke, CHF, and any CVD (either ACS, stroke, or CHF) were identified from HDR using the same criteria as for the pre-existing events. Repeat revascularizations were considered with the same NOMESCO codes as defined above.

### Statistical methods

The data were pooled from all first cardiac revascularisation procedures performed between the years 2000 and 2015. Baseline variables were summarized using descriptive statistics presented as means and standard deviations (SDs). Comparisons of the baseline characteristics were performed using Student's *t*-test for continuous variables, and chi-square test for categorical variables.

Kaplan–Meier curves and log-rank tests were used for comparing event-free survivals after the first revascularisation procedure between diabetic and non-diabetic patients. Similarly, event-free survivals among diabetic patients were compared between those who had PCI and those who had CABG as the revascularisation method. The same comparison was repeated for non-diabetic patients. Cox proportional hazards regression analyses were used for computing hazard ratios (HRs) of outcome events comparing different revascularisation methods, i.e. PCI with CABG. The multivariate model was adjusted for age, gender, year of procedure, region of residence, valvular defects, ACS/CHD/cardiomyopathy, hypertension, stroke, atrial fibrillation, peripheral arterial disease, and duration of diabetes. These analyses were carried out separately for diabetic and non-diabetic patients. Schoenfeldt residuals were used to ascertain validity of the proportional hazards assumption.

We considered *P* < 0.05 to be statistically significant for all analyses. When appropriate, 95% confidence intervals (CIs) are presented. All statistical analyses were carried out using R statistical software version 3.6.0 (R Core Team, 2019).

## Results

### Patient characteristics and overall event-free survival

A total of 139 242 patients were revascularised during the study period. PCI was more common than CABG as a revascularisation procedure both among patients with and without diabetes, in both genders and all comorbidity subgroups (*Table 1*). Altogether 49 749 CABG (35.7% of total revascularisations) and 89 493 PCI (64.3%) procedures were performed. Among all revascularisations, men accounted for a total of 99 437 (71.4%) cases and women for 39 805 (28.6%). Urgent procedures constituted 59 224 (42.5%) of all revascularisations. Of the revascularised patients, 33 018 (23.7%) had diabetes, whereas 106 224 (76.3%) did not. Coronary artery bypass grafting constituted 39.1% of all revascularisations among patients with diabetes and 34.7% among non-diabetic patients. Patients with diabetes were slightly older compared to non-diabetic patients and presented more often with a history of previous ACS and stroke, and had a higher prevalence of CHF and hypertension than non-diabetic patients (*Table 1*).

**Table 1:** Patient characteristics at the study baseline

	DM	Non-diabetic	P*
N	33 018	106 224	
Age	68.2 (10.1)	66.4 (11.0)	<0.0001
Women	10 647 (32.2)	29 158 (27.4)	<0.0001
Urgent procedure	13 591 (41.2)	45 633 (43.0)	<0.0001
Previous MI	9603 (29.1)	22 714 (21.4)	<0.0001
Previous stroke	3044 (9.2)	5496 (5.2)	<0.0001
CHF	19 858 (60.1)	61 588 (58.0)	<0.0001
Hypertension	17 038 (51.6)	32 679 (30.8)	<0.0001
ACS/CHD/Cardiomyopathy	26 943 (81.6)	83 936 (79.0)	<0.0001
AF	4050 (12.3)	9078 (8.5)	<0.0001
Valvular deficiency	2661 (8.1)	7584 (7.1)	<0.0001
PAD	2932 (8.9)	3659 (3.4)	<0.0001
Duration of diabetes	7.8 (6.0)	N/A	N/A
**CABG**			
N	12 900	36 849	
Age	67.6 (9.1)	67.2 (9.6)	0.0003
Women	3638 (28.2)	8675 (23.5)	<0.0001
Urgent procedure	3763 (29.2)	10 271 (27.9)	<0.0001
Previous MI	4593 (35.6)	11 121 (30.2)	<0.0001
Previous stroke	1183 (9.2)	2164 (5.9)	<0.0001
CHF	6891 (53.4)	16 887 (45.8)	<0.0001
Hypertension	6279 (48.7)	11 044 (30.0)	<0.0001
ACS/CHD/Cardiomyopathy	9727 (75.4)	25 536 (69.3)	<0.0001
AF	1351 (10.5)	3084 (8.4)	<0.0001
Valvular deficiency	1732 (13.4)	5289 (14.4)	0.0050
PAD	1232 (9.6)	1549 (4.2)	<0.0001
Duration of diabetes	7.4 (5.6)	N/A	N/A
**PCI**			
N	20 118	69 375	
Age	68.6 (10.7)	65.9 (11.6)	<0.0001
Women	7009 (34.8)	20 483 (29.5)	<0.0001
Urgent procedure	9828 (48.9)	35 362 (51.0)	<0.0001
Previous MI	5010 (24.9)	11 593 (16.7)	<0.0001
Previous stroke	1861 (9.3)	3332 (4.8)	<0.0001
CHF	12 967 (64.5)	44 701 (64.4)	<0.0001
Hypertension	10 759 (53.5)	21 635 (31.2)	<0.0001
ACS/CHD/Cardiomyopathy	17 216 (85.6)	58 400 (84.2)	<0.0001
AF	2699 (13.4)	5994 (8.6)	<0.0001
Valvular deficiency	938 (4.7)	2295 (3.3)	<0.0001
PAD	1700 (8.5)	2110 (3.0)	<0.0001
Duration of diabetes	8.0 (6.2)	N/A	N/A

Numbers are mean (±sd) for age and duration of diabetes in years, n (%) for other variables. AF, atrial fibrillation; ACS, acute coronary syndrome; CABG, coronary artery bypass surgery; CHD, coronary heart disease; CHF, chronic heart failure; DM, diabetes mellitus (including both type I and II); MI, myocardial infarction; PAD, peripheral artery diseases; PCI, percutaneous coronary intervention. Procedure was considered urgent if performed during 7 days after hospitalization. Groups were compared using *t*-test for continuous variables and chi-square test for categorical variables.

* Statistically significant at p<0.05.

**Figure 1:**
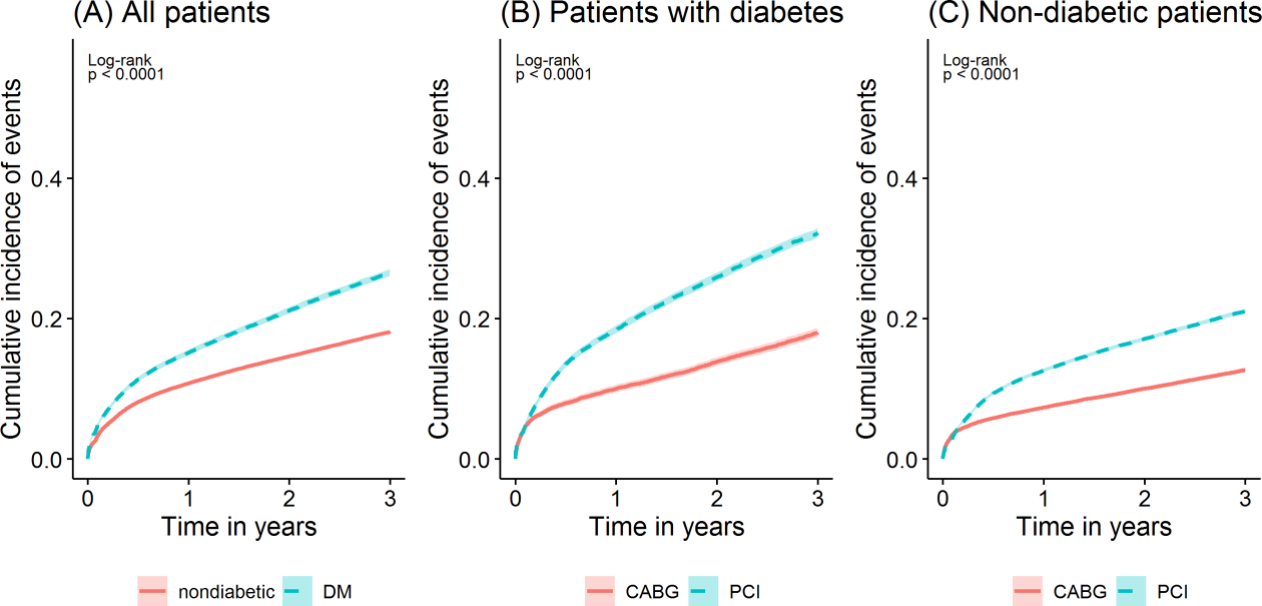
Kaplan–Meier estimates of cumulative incidences of composite outcome event of all-cause death, any cardiovascular event, and repeat revascularisation procedures. The cumulative incidences among (A) diabetic and non-diabetic patients for all revascularisation procedures, and in (B) diabetic patients and (C) non-diabetic patients for coronary artery bypass grafting and percutaneous coronary intervention separately. P-values are for log-rank tests, statistically significant if *P* < 0.05.

When examining the whole 3-year period after the first revascularisation procedure using Kaplan–Meier curves, patients with diabetes had lower event-free survival compared to non-diabetic patients when the data from both CABG and PCI were pooled (*Figure 1A*). When comparing the two revascularisation methods over the whole 3 year period for all outcome events, event-free survival of patients treated with CABG was better than those treated with PCI in both diabetic and non-diabetic patients (*Figure 1B, C*).

### Short-term outcomes

The numbers of different outcome events at 28 days among diabetic and non-diabetic patients grouped by CABG and PCI are shown in detail in *Table 2*. In multivariate adjusted analyses, non-diabetic patients treated with PCI had lower risk of all-cause deaths and CVD deaths when compared to patients treated with CABG, whereas no significant differences were observed between the two revascularisation methods among patients with diabetes. Both among patients with diabetes and non-diabetic patients, PCI was associated with lower risk for all other outcome events when compared to CABG except for repeat revascularisation procedures, where CABG was associated with lower risk in both patient groups (*Table 3*).

**Table 2:** Crude incident outcome event numbers and percentages after revascularization at the baseline in diabetic (PCI n = 20 118; CABG n = 12 900; total n = 33 018) and non-diabetic (PCI n = 69 375; CABG n = 36 849; total n = 106 224) patients grouped by revascularisation procedure in the time points of 28 days, 1 year and 3 years^a^

	28 days			1 year			3 year		
	PCI	CABG	Total	PCI	CABG	Total	PCI	CABG	Total
All-cause deaths									
Non-diabetic	1534 (2.2%)	1023 (2.8%)	2557 (2.4%)	3253 (4.7%)	1808 (4.9%)	5061 (4.8%)	6052 (8.7%)	3072 (8.3%)	9124 (8.6%)
Diabetic	736 (3.7%)	462 (3.6%)	1198 (3.6%)	1764 (8.8%)	925 (7.2%)	2689 (8.1%)	3373 (16.8%)	1616 (12.5%)	4989 (15.1%)
Cardiovascular deaths									
Non-diabetic	1487 (2.1%)	991 (2.7%)	2478 (2.3%)	2849 (4.1%)	1655 (4.5%)	4504 (4.2%)	4847 (7.0%)	2570 (7.0%)	7417 (7.0%)
DM	712 (3.5%)	446 (3.5%)	1158 (3.5%)	1588 (7.9%)	845 (6.6%)	2433 (7.4%)	2850 (14.2%)	1369 (10.6%)	4219 (12.8%)
Acute coronary syndrome									
Non-diabetic	126 (0.3%)	183 (0.5%)	309 (0.3%)	979 (1.4%)	580 (1.6%)	1559 (1.5%)	1913 (2.8%)	976 (2.6%)	2889 (2.7%)
Diabetic	58 (0.3%)	84 (0.7%)	142 (0.4%)	494 (2.5%)	252 (2.0%)	746 (2.3%)	1006 (5.0%)	494 (3.8%)	1500 (4.5%)
Stroke									
Non-diabetic	15 (0.02%)	48 (0.1%)	63 (0.1%)	518 (0.7%)	418 (1.1%)	936 (0.9%)	1376 (2.0%)	954 (2.6%)	2330 (2.2%)
Diabetic	6 (0.03%)	18 (0.1%)	24 (0.1%)	231 (1.1%)	183 (1.4%)	414 (1.3%)	597 (3.0%)	414 (3.2%)	1011 (3.1%)
Any CVD^b^									
Non-diabetic	172 (0.2%)	212 (0.6%)	384 (0.4%)	1176 (1.7%)	809 (2.2%)	1985 (1.9%)	2290 (3.3%)	1442 (3.9%)	3732 (3.5%)
Diabetic	66 (0.3%)	89 (0.7%)	155 (0.5%)	490 (2.4%)	340 (2.6%)	830 (2.5%)	1020 (5.1%)	627 (4.9%)	1647 (5.0%)
Heart failure									
Non-diabetic	188 (0.3%)	221 (0.6%)	409 (0.4%)	1101 (1.6%)	701 (1.9%)	1802 (1.7%)	2055 (3.0%)	1127 (3.1%)	3182 (3.0%)
Diabetic	71 (0.4%)	102 (0.8%)	173 (0.5%)	497 (2.5%)	306 (2.4%)	803 (2.4%)	983 (4.9%)	537 (4.2%)	1520 (4.6%)
Repeat revascularisation									
Non-diabetic	104 (0.1%)	20 (0.1%)	124 (0.1%)	4755 (6.9%)	173 (0.5%)	4928 (4.6%)	6863 (9.9%)	289 (0.8%)	7152 (6.7%)
Diabetic	41 (0.2%)	6 (0.05%)	47 (0.1%)	1687 (8.4%)	80 (0.6%)	1767 (5.4%)	2537 (12.6%)	133 (1.0%)	2670 (8.1%)

^a^All-cause mortality and cardiovascular mortality are without exclusions. For the other outcomes, persons with the same event prior to baseline have been excluded from the analyses. CABG, coronary artery bypass surgery; CVD, cardiovascular disease event; Diabetic patients (including both type I and II); PCI, percutaneous coronary intervention.

^b^Any CVD defined as an incident event of any of the following: ACS, stroke, or heart failure (excluding cases with any prevalent CVD, either ACS, stroke, or heart failure, before baseline).

**Table 3:** Hazard ratios (PCI vs. CABG) for revascularised diabetic and non-diabetic patients for the outcome events in the time points of 28 days, 1 year, and 3 years^a^

	28 days		1 year		3 year	
	HR (95% CI)^b^	P*	HR (95% CI)^b^	P*	HR (95% CI)^b^	P*
All-cause deaths						
Non-diabetic	0.75 (0.69–0.81)	<0.001	0.94 (0.88–1.00)	0.050	1.09 (1.04–1.15)	<0.001
DM	0.89 (0.78–1.00)	0.059	1.11 (1.02–1.20)	0.018	1.30 (1.22–1.38)	<0.001
Cardiovascular deaths						
Non-diabetic	0.74 (0.68–0.81)	<0.001	0.89 (0.83–0.95)	<0.001	1.03 (0.98–1.08)	0.260
DM	0.90 (0.79–1.02)	0.086	1.10 (1.00–1.20)	0.041	1.29 (1.20–1.38)	<0.001
Acute coronary syndrome						
Non-diabetic	0.33 (0.26–0.42)	<0.001	0.99 (0.88–1.11)	0.825	1.21 (1.11–1.32)	<0.001
DM	0.39 (0.27–0.56)	<0.001	1.27 (1.08–1.50)	0.004	1.36 (1.21–1.53)	<0.001
Stroke						
Non-diabetic	0.19 (0.10–0.35)	<0.001	0.71 (0.62–0.82)	<0.001	0.86 (0.78–0.94)	0.001
DM	0.20 (0.08–0.52)	0.001	0.84 (0.69–1.03)	0.103	1.00 (0.88–1.14)	0.980
Any CVD						
Non-diabetic	0.63 (0.51–0.78)	<0.001	1.27 (1.15–1.40)	<0.001	1.45 (1.35–1.56)	<0.001
DM	0.60 (0.43–0.84)	0.003	1.30 (1.12–1.51)	<0.001	1.51 (1.36–1.68)	<0.001
Chronic heart failure						
Non-diabetic	0.66 (0.54–0.82)	<0.001	1.30 (1.18–1.44)	<0.001	1.67 (1.54–1.80)	<0.001
DM	0.56 (0.41–0.77)	<0.001	1.35 (1.16–1.56)	<0.001	1.71 (1.53–1.91)	<0.001
Repeat revascularisation^c^						
Non-diabetic	2.36 (1.44–3.89)	0.001	15.32 (13.14–17.86)	<0.001	13.91 (12.34–15.68)	<0.001
DM	4.47 (1.85–10.77)	0.001	14.27 (11.38–17.91)	<0.001	13.50 (11.32–16.11)	<0.001

^a^Outcome events were all-cause deaths, cardiovascular deaths, acute coronary syndromes (ACSs), stroke, any cardiovascular event (CVD), chronic heart failure (CHF), re-operation either CABG (coronary artery bypass surgery) or new PCI (percutaneous coronary intervention). All-cause mortality and cardiovascular mortality are without exclusions. For the other outcomes, persons with the same event prior to baseline have been excluded from the analyses.

^b^Numbers are hazard ratios (HRs) with 95% confidence intervals (CIs) shown in parentheses. The HR model includes adjustments for age, gender, year of procedure, region of residence, valvular defects, ACS/CHD/cardiomyopathy, hypertension, previous stroke, atrial fibrillation, peripheral arterial disease, and duration of diabetes.

^c^Repeat revascularisations during the 28 days period were excluded from the analysis of the repeat revascularisations at 1 and 3 year time points.

DM: diabetes mellitus (including both type I and II).

*P-values significant at the level of *P* < 0.05.

### Fatal long-term outcome events

A total of 7750 and 14 113 all-cause deaths were observed at 1 year and 3 year time points, respectively (*Table 2*). The fatal long-term outcomes after CABG and PCI differed between the diabetic and non-diabetic groups. When evaluated at the time point of 1 year, PCI was associated with higher risk for all-cause deaths than CABG among diabetic patients, whereas no difference was observed between the revascularisation methods among non-diabetic patients (*Table 3*). At the 3 year time point, however, PCI was associated with higher risk for all-cause deaths than CABG in both diabetic and non-diabetic patients. Moreover, the excess risk in the PCI group was even higher among diabetic patients than among non-diabetic patients (*Table 3*). Correspondingly, there were differences in the long-term incidence of cardiovascular death by diabetes status. At the 1 year time point, non-diabetic patients treated with PCI had lower risk of CVD death than those treated with CABG. However, at the 3 year time point, there were no longer significant differences between PCI and CABG among the non-diabetic patients. Conversely, patients with diabetes treated with CABG had lower risk of CVD death than those treated with PCI at both the 1 year and 3 year time points (*Table 3*).

We analysed further the fatal long-term outcomes for those subjects, who had remained event-free for 28 days following the first revascularisation procedure. Altogether, 31 657 (95.9%) diabetic patients and 103 229 (97.2%) non-diabetic patients had an event-free 28 day survival. Among these patients, CABG was associated with lower risk for all-cause and cardiovascular death when compared to PCI both at 1 year and 3 year time points in diabetic as well as in and non-diabetic patients. At the 3 year time point, the risk of CVD death after PCI when compared to CABG was more elevated in patients with diabetes than in non-diabetic patients (*Table 4*).

**Table 4:** Hazard ratios, PCI vs. CABG, for the outcome events^a^ in the time points of 1 year and 3 years among revascularised diabetic and non-diabetic patients with event-free survival for the first 28 days

	1-year		3-year	
	HR (95% CI)^b^	P*	HR (95% CI)^b^	P*
All-cause deaths				
Non-diabetic	1.22 (1.11–1.33)	<0.001	1.29 (1.22–1.37)	<0.001
DM	1.33 (1.18–1.49)	<0.001	1.47 (1.37–1.58)	<0.001
Cardiovascular deaths				
Non-diabetic	1.13 (1.02–1.25)	0.018	1.24 (1.16–1.32)	<0.001
DM	1.32 (1.17–1.50)	<0.001	1.48 (1.37–1.61)	<0.001
Acute coronary syndrome				
Non-diabetic	1.34 (1.17–1.53)	<0.001	1.45 (1.32–1.59)	<0.001
DM	1.72 (1.42–2.08)	<0.001	1.56 (1.38–1.77)	<0.001
Stroke				
Non-diabetic	0.78 (0.68–0.90)	0.001	0.89 (0.81–0.98)	0.013
DM	0.92 (0.74–1.14)	0.432	1.04 (0.91–1.19)	0.606
Any CVD				
Non-diabetic	1.51 (1.36–1.69)	<0.001	1.61 (1.49–1.73)	<0.001
DM	1.58 (1.34–1.87)	<0.001	1.68 (1.50–1.88)	<0.001
Chronic heart failure				
Non-diabetic	1.73 (1.53–1.94)	<0.001	1.94 (1.78–2.11)	<0.001
DM	1.96 (1.64–2.34)	<0.001	2.00 (1.77–2.26)	<0.001

^a^Outcome events were all-cause deaths, cardiovascular deaths, acute coronary syndromes (ACSs), stroke, any cardiovascular event (CVD), and chronic heart failure (CHF). All-cause mortality and cardiovascular mortality are without exclusions. For the other outcomes, persons with the same event prior to baseline have been excluded from the analyses.

^b^Numbers are hazard ratios (HRs) with 95% confidence intervals (CIs) shown in parentheses. The HR model includes adjustments for age, gender, year of procedure, region of residence, valvular defects, ACS/CHD/cardiomyopathy, hypertension, previous stroke, atrial fibrillation, peripheral arterial disease, and duration of diabetes.

*P-values significant at the level of *P* < 0.05.

DM, diabetes mellitus (including both type I and II).

### Cause specific long-term outcome events

When evaluating the risk for other outcome events, including non-fatal outcomes, at 1 and 3 year time points from the revascularisation, the risk for CHF and any CVD event was significantly lower after CABG than PCI in both diabetic and non-diabetic patients. There were no differences in these risks between the diabetic and non-diabetic patients (*Table 3*). Similarly, repeat revascularisation procedures were significantly less frequent after CABG than after PCI in both patient groups at the 1 year and 3 year time points. On the other hand, the risk for stroke was significantly lower after PCI compared to CABG in non-diabetic patients at both 1 and 3 year time points; whereas among patients with diabetes, no differences were observed in the long-term stroke risk between the revascularisation methods in neither of these time points (*Table 3*). Regarding the risk for ACS at the 1 year time point, CABG was associated with lower risk for ACS than PCI among diabetic patients, whereas no difference was observed in non-diabetic patients (*Table 3*). However, at 3 years, the risk for ACS was lower after CABG when compared to PCI among both patient groups (*Table 3*).

When we analyzed patients with event-free survival for the first 28 days at the 1 year and 3 year time points, we observed lower risk of ACS, heart failure, and any CVD event after CABG than PCI among both diabetic and non-diabetic patients (*Table 4*).

## Discussion

Our large register-based study reflecting real-world clinical practice showed that the long-term prognosis was better with CABG than with PCI in both non-diabetic and diabetic patients. At the 3 year time point, CABG was associated with lower risk for all-cause death compared to PCI in both non-diabetic and diabetic patients. Coronary artery bypass grafting was also associated with lower long-term risk for CVD death among patients with diabetes at 3 years. As for non-diabetic patients in regard to the fatal CVD outcomes, CABG was associated with better prognosis than PCI at 3 years among those individuals, who had remained event-free for the first 28 days. Importantly, the better prognosis after CABG than after PCI regarding these long-term fatal CVD outcomes was even more evident among diabetic patients than among non-diabetic patients. For those patients who had remained event free for the first 28 days after revascularisation, CABG was associated with lower risk for CHF and all cardiovascular events, except for stroke among patients with diabetes, when compared to PCI. Regarding all fatal outcomes, the short-term prognosis at 28 days was better after PCI than after CABG in non-diabetic patients, whereas no difference was observed between the methods among patients with diabetes. However, the short-term prognosis was better after PCI than CABG for all other cause-specific outcomes in both patient groups except for the need of repeat revascularisations.

The better long-term outcomes after CABG compared to PCI in our study are in line with another observational study (ASCERT), which reported an adjusted 4 year mortality of 16.4% for CABG and of 20.8% for PCI with first-generation DESs among patients aged 65 or older, and an adjusted risk ratio favoring CABG both in non-diabetic and diabetic patients.^23^ Our real-life results on non-selected patients are also consistent with the findings on diabetic patients with multivessel disease in original RCT studies^9–11^ and pooled analyses of RCT studies.^16^ FREEDOM trial, the first large RCT on revascularisation methods for diabetic patients published in 2012, compared PCI with DESs and CABG among diabetic patients with stable-ischemic multivessel CHD. This study showed that in a 3.8-year follow-up, CABG was superior to PCI, with all-cause mortality among patients with CABG being 10.9% compared to 16.3% in patients with PCI (*P* = 0.049).^10^ These results were further confirmed after 7.5 years of follow-up, where all-cause mortality after CABG was 18.3% compared to 24.3% after PCI (*P* = 0.01).^11^ Better long-term survival after CABG compared to PCI was confirmed also by a pooled analysis in a systematic review (powered to detect all-cause mortality), which reported a 10.0% all-cause mortality after a 5 year follow-up among diabetic patients with multivessel disease treated with CABG compared to 15.5% in patients treated with PCI (with either BMS or DES). In contrast to our results, this pooled analysis did not find differences in all-cause mortality among non-diabetic patients.^16^

Interestingly, we found no differences in the risk for fatal all-cause and CVD deaths in 28 days between the revascularisation methods among diabetic patients, whereas non-diabetic patients who underwent PCI had better short-term prognosis when compared to CABG. Similar to our results, the FREEDOM study^10^ showed no differences in the short-term CVD mortality between PCI and CABG in diabetic patients. However, on the contrary to the results in the FREEDOM study, we showed better short-term prognosis regarding the risk for ACS and stroke after PCI when compared to CABG, both in diabetic and non-diabetic patients; whereas the FREEDOM study^10^ showed no differences in the risk for major cardiovascular and cerebrovascular events. This discrepancy may be explained by differences in patient selection in real-world clinical practice when compared to RCTs. In observational studies, differences have been observed in the characteristics of the patients about to undergo either CABG or PCI. On average, patients treated with PCI have been shown to be older and more often women, whereas patients selected for CABG have more often generalized and 2-3 vessel disease.^23^

However, for the long-term prognosis, it may be that patient selection does not fully explain the outcome differences: Tam *et al.*^24^ examined outcomes after CABG and PCI using clinical and administrative databases in Canada, and performed a 1:1 propensity score matching of 23 baseline characteristics for 4301 pairs of patients with diabetes and 2–3 vessel CHD. After matching for comorbidities, CABG was associated with lower mortality and lower risk for major adverse cardiac and cardiovascular events (MACCEs) when compared to PCI in 8 year follow-up.^24^ In addition, different mechanism of achieving the revascularisation has been suggested to explain the superiority of CABG over PCI. Namely, CABG may provide additional protection from those lesions that are non-flow limiting at the time of the procedure, and thus not treated with PCI but bypassed with CABG and resulting in a wider area with collateral circulation for CABG compared to PCI.^25^ It could also be speculated that differences in the long-term outcomes between revascularisation methods could arise from differences in secondary prevention after revascularisation. However, evidence suggests that the differences herein would be in favor of PCI, and thus, not explaining the differences in the outcomes. In the FREEDOM study, controlled optimal medical treatment was provided after both PCI and CABG.^10^ In addition, observational studies have shown that patients who had undergone CABG filled fewer prescriptions for statins, ACE inhibitors, and ARB blockers compared to those patients for whom PCI was performed.^26^ Moreover, compliance with guideline-directed medical therapy after revascularisation has been shown to be worse after CABG than PCI in clinical trials.^27^ Further studies are needed to clarify whether these findings apply also to revascularised patients with diabetes.

Some criticism has also been raised against the outcome measures used in revascularisation trials. A recently published meta-analysis concluded that PCI was associated with higher all-cause, cardiac, and non-cardiac mortality when compared to CABG at 5 years.^28^ Due to the higher non-cardiac mortality with PCI, the authors suggested that all-cause mortality should be used as the primary end point for revascularisation trials in CHD. Our overall results of the lower all-cause long-term mortality after CABG when compared to PCI are in line with the meta-analysis by Gaudino *et al.*^28^

In our previous study, we have shown that only approximately 27% of diabetic patients and 22% of non-diabetic patients in Finland in 2012–2015 had received CABG as their first revascularisation procedure.^21^ This may imply that some of the patients eligible for CABG might have received PCI instead. Since, as shown in this study, the long-term prognosis seems to be better after CABG especially for patients with diabetes; a balanced evaluation of the optimal revascularisation strategy for an individual patient, performed by a multidisciplinary heart team, would be vital for achieving the best individual outcome.^20^

### Strengths and limitations

The major strength in our study is that due to the mandatory documentation protocols, the Finnish HDR covers all hospitalisations and nearly all revascularisations in Finland including the follow-up.^29^ We were also able to identify all known diabetic patients by using the HDR and DRR. With these registers we identified the important comorbidities.

We also acknowledge limitations in our study. Firstly, we did not have access to individual patient data depicting the clinical characteristics and detailed clinical situation leading to revascularisation or concurrent medications, nor data on the extent of the coronary atherosclerosis. We had neither access to the data on the types of the stents used in PCI nor on the detailed descriptions of the bypass technique in CABG.

## Conclusions

Although PCI was associated with better or non-different short-term prognosis, CABG was associated with better long-term prognosis especially among patients with diabetes at the 3 year time point. Our results provide further support to earlier findings suggesting better long-term prognosis after CABG than after PCI in diabetic patients. However, further randomized trials are warranted to determine in more detail the clinical characteristics that influence the choice of the revascularization method in diabetic individuals.

## Supplementary Material

qcab065_Supplemental_Files

## Notes

**Conflict of interest: KW** has received honoraria from Novo Nordisk and AstraZeneca for lecturing, and from Sanofi for consulting, all unrelated to the present study. **JL** has received honoraria from Bayer Ltd. and Boehringer Ingelheim for lecturing. **VS** has received honoraria from Novo Nordisk and Sanofi for consulting. He also has ongoing research collaboration with Bayer Ltd. (all unrelated to the present study). **H-R L, AP, and TN have** none to declare.

## Data availability

Due to the privacy and legal restrictions, individual level data used in this article are not available.
