# Supplementary material for: Outcomes after coronary artery bypass grafting and percutaneous coronary intervention in diabetic and non-diabetic patients
Source: Eur Heart J Qual Care Clin Outcomes. 2021 Sep 7;8(6):692–700. doi: 10.1093/ehjqcco/qcab065 (PMC10027652; doi:10.1093/ehjqcco/qcab065)
Supplement: qcab065_Supplemental_Files [file qcab065_supplemental_files.zip › Supplementary picture_1_28.8.docx]

Supplementary picture 1

All first revascularizations in 2000-2015

n=139, 242

Diabetic

12,900

Diabetic

20,118

Registry follow-up for all-cause mortality and cardiovascular outcomes at 28-day, 1-year and 3-year time points

CABG

49,749

Non-diabetic

36,849

PCI

89,493

Non-diabetic

69,375
