# Supplementary material for: Outcomes after coronary artery bypass grafting and percutaneous coronary intervention in diabetic and non-diabetic patients
Source: Eur Heart J Qual Care Clin Outcomes. 2021 Sep 7;8(6):692–700. doi: 10.1093/ehjqcco/qcab065 (PMC10027652; doi:10.1093/ehjqcco/qcab065)
Supplement: qcab065_Supplemental_Files [file qcab065_supplemental_files.zip › Supplementary table 1_29.8.2021.docx]

**Supplementary table 1:** Clinical definitions of the used NOMESCO Nordic- Medico- Statistical Committee codes

| **CABG** |  |
| --- | --- |
| FNA | Connection to coronary artery from internal mammary artery |
| FNB | Connection to coronary artery from gastroepiploic artery |
| FNC | Aorto- coronary venous bypass |
| FND | Aorto- coronary bypass using prosthetic graft |
| FNE | Coronary bypass using free arterial graft |
| **PCI** |  |
| FN1AT | Endovascular dilatation of coronary arteries (PTCA) |
| FN1BT | Extensive endovascular dilatation of coronary (PTCA) arteries |
| FN1YT | Percutaneous insertion of coronary artery stent |
| FNF | Coronary thrombendarterectomy |
| FNG | Recanalisation of coronary artery |
| TFN40 | Catheterisation of heart with balloon widening of coronary vessels |
