# Supplementary material for: Outcomes after coronary artery bypass grafting and percutaneous coronary intervention in diabetic and non-diabetic patients
Source: Eur Heart J Qual Care Clin Outcomes. 2021 Sep 7;8(6):692–700. doi: 10.1093/ehjqcco/qcab065 (PMC10027652; doi:10.1093/ehjqcco/qcab065)
Supplement: qcab065_Supplemental_Files [file qcab065_supplemental_files.zip › Supplementary table 2_29.8.docx]

**Supplementary table 2:** Clinical definitions of the used ICD (International Classification of Diseases) -codes

| E10 | Type 1 diabetes mellitus |
| --- | --- |
| E11 | Type 2 diabetes mellitus |
| I10 | Essential (primary) hypertension |
| I11 | Hypertensive heart disease |
| I12 | Hypertensive chronic kidney disease |
| I13 | Hypertensive heart and chronic kidney disease |
| I15 | Secondary hypertension |
| I50 | Heart failure |
| I20 | Ischemic heart diseases |
| I21 | Acute myocardial infarction |
| I22 | Subsequent ST elevation (STEMI) and non-ST elevation (NSTEMI) myocardial infarction |
| I23 | Certain current complications following ST elevation (STEMI) and non-ST elevation (NSTEMI) myocardial infarction (within the 28 day period) |
| I24 | Other acute ischemic heart diseases |
| I25 | Chronic ischemic heart disease |
| I42 | Cardiomyopathy |
| I43 | Cardiomyopathy in diseases classified elsewhere |
| I05 | Rheumatic mitral valve diseases |
| I06 | Rheumatic aortic valve diseases |
| I07 | Rheumatic tricuspid valve diseases |
| I08 | Multiple valve diseases |
| I34 | Nonrheumatic mitral valve disorders |
| I35 | Nonrheumatic aortic valve disorders |
| I36 | Nonrheumatic tricuspid valve disorders |
| I37 | Nonrheumatic pulmonary valve disorders |
| I39.0 | Mitral valve disorders in diseases classified elsewhere |
| I39.1 | Aortic valve disorders in diseases classified elsewhere |
| I39.2 | Tricuspid valve disorders in diseases classified elsewhere |
| I39.3 | Pulmonary valve disorders in diseases classified elsewhere |
| I39.4 | Multiple valve disorders in diseases classified elsewhere |
| I48 | Atrial fibrillation and flutter |
| I61 | Nontraumatic intracerebral hemorrhage |
| I62 | Other and unspecified nontraumatic intracranial hemorrhage |
| I63 | Cerebral infarction |
| I64 | Stroke, not specified as haemorrhage or infarction |
| I70.2 | Atherosclerosis of native arteries of the extremities |
| R96 | Instantaneous death |
| R98 | Unattended death |
